# Genomic data reveal a loss of diversity in two species of tuco-tucos (genus *Ctenomys*) following a volcanic eruption

**DOI:** 10.1038/s41598-017-16430-1

**Published:** 2017-11-24

**Authors:** Jeremy L. Hsu, Jeremy Chase Crawford, Mauro N. Tammone, Uma Ramakrishnan, Eileen A. Lacey, Elizabeth A. Hadly

**Affiliations:** 1 Department of Biology, Stanford University, Stanford, CA USA; 2 Museum of Vertebrate Zoology and Department of Integrative Biology, University of California, Berkeley, CA USA; 3 Programa de Estudios Aplicados a la Conservación (CENAC-PNNH, CONICET), Bariloche, Río Negro, Argentina; 4 National Centre for Biological Sciences, Bangalore, India; 5 Senior Fellow, Woods Institute for the Environment, Stanford University, Stanford, CA USA; 6 Senior Fellow, Center for Innovation in Global Health, Stanford University, Stanford, CA USA; 7 Present Address: Chapman University, 92866 Orange, CA USA

## Abstract

Marked reductions in population size can trigger corresponding declines in genetic variation. Understanding the precise genetic consequences of such reductions, however, is often challenging due to the absence of robust pre- and post-reduction datasets. Here, we use heterochronous genomic data from samples obtained before and immediately after the 2011 eruption of the Puyehue-Cordón Caulle volcanic complex in Patagonia to explore the genetic impacts of this event on two parapatric species of rodents, the colonial tuco-tuco (*Ctenomys sociabilis*) and the Patagonian tuco-tuco (*C. haigi*). Previous analyses using microsatellites revealed no post-eruption changes in genetic variation in *C. haigi*, but an unexpected increase in variation in *C. sociabilis*. To explore this outcome further, we used targeted gene capture to sequence over 2,000 putatively neutral regions for both species. Our data revealed that, contrary to the microsatellite analyses, the eruption was associated with a small but significant decrease in genetic variation in both species. We suggest that genome-level analyses provide greater power than traditional molecular markers to detect the genetic consequences of population size changes, particularly changes that are recent, short-term, or modest in size. Consequently, genomic analyses promise to generate important new insights into the effects of specific environmental events on demography and genetic variation.

## Introduction

Significant reductions in population size (i.e. population bottlenecks^1^) can substantially impact patterns of genetic variation and thus potentially alter the evolutionary trajectories of affected organisms. Accordingly, the genetic consequences of bottlenecks have been the focus of numerous empirical and theoretical studies (e.g.^1–6^). Such consequences can be difficult to assess, however, particularly when reductions in population size are modest in magnitude or duration. In part, this difficulty reflects the frequent use of traditional molecular markers such as microsatellites or mitochondrial loci, both of which may have limited power to detect changes in genetic variation over small spatial or temporal scales^2,3^. In contrast, examination of much larger numbers of markers drawn from across the genome should increase the ability to detect temporal and spatial differences in genetic variation^4,5^.

Although use of genomic data to address evolutionary questions is increasing rapidly, only a limited number of studies have employed such information to study the genetic consequences of naturally occurring demographic bottlenecks. Instead, most analyses that have used genomic data to assess the impacts of reductions in population size have focused on demographic changes associated with agricultural activities such as domestication of plants and animals^3,6–8^. The relevance of these analyses to wild populations is unclear, as domesticated species have typically been subject to strong artificial selection that may confound signals of other evolutionary forces such as drift, mutation, migration, and gene flow^3^. Further, these studies have typically relied on samples taken at only a single point in time, often many generations after domestication, which may alter the ability to detect changes in genetic variation relative to studies based on samples collected shortly before and after a demographic event^9,10^. Thus, while genomic analyses appear to offer considerable potential, their suitability for studies of naturally occurring changes in genetic variation – particularly those occurring over relatively short time periods – remains largely unexplored.

To evaluate the immediate genetic consequences of abrupt, naturally occurring reductions in population size, we generated a genome-wide panel of single-nucleotide polymorphisms for two species of ctenomyid rodents from the Limay Valley region of southwestern Argentina. The colonial tuco-tuco (*Ctenomys sociabilis*) and the parapatric Patagonian tuco-tuco (*C. haigi*) have been the subjects of extensive behavioral, ecological, demographic, and genetic research, including comparative analyses of genetic variation over multiple spatial and temporal scales^11–15^. In 2011, both species were significantly affected by the eruption of the Puyehue-Cordón Caulle volcanic complex in southern Chile, resulting in deceases in population density of ~40% for *C. sociabilis* and ~25% for *C. haigi* ^16^ and providing a rare opportunity to compare directly levels of genetic variation before and after a natural population decline. Notably, previous analyses based on microsatellite markers^16^ suggested that this event was associated with a significant post-eruption increase in genetic variation in *C. sociabilis* but not in *C. haigi*.

To explore this unexpected outcome in greater detail, we employed targeted sequence capture to generate a large number of genome-wide markers for each study species. Specifically, we sought to determine if the expected greater resolution of these genomic analyses would reveal the same post-eruption increase in genetic variation in *C. sociabilis*. Additionally, we sought to identify potential signatures of demographic processes contributing to post-eruption changes in genetic variation in our study species. While multiple studies have explored post-bottleneck changes in demography over longer time scales (e.g.^10,17–21^), the more immediate impacts of these demographic parameters are not well characterized. As a result, our analyses of the effects of the Puyehue-Cordón Caulle eruption should generate important new insights into the role of demographic processes in shaping short-term genomic responses to pronounced reductions in population size.

## Materials and Methods

### Study system

**Figure 1:**
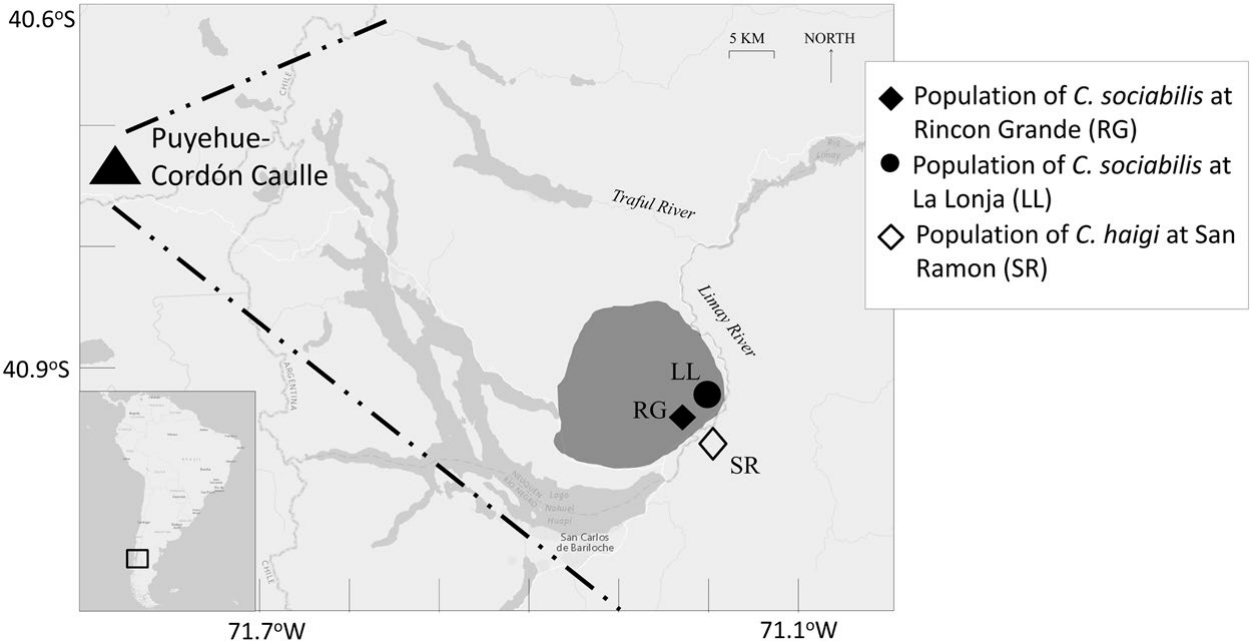
Map of study area in southwestern Argentina. The general cone of ash fall resulting from the 2011 eruption is indicated with the dashed lines. The geographic distribution of *C. sociabilis* is indicated in dark gray. Figure modified with permission from Hsu *et al*.^16^.

The two species of tuco-tucos (genus *Ctenomys*) studied have been the subjects of long-term field research on behavior, ecology, and demography. The colonial tuco-tuco (*Ctenomys sociabilis*) is endemic to an approximately 1000 square kilometer area in the western Limay Valley and adjacent hills of Neuquén Province, Argentina (Fig. 1;^22,23^). In contrast, the Patagonian tuco-tuco (*C. haigi*) is much more widely distributed in the eastern Limay Valley and surrounding regions of Río Negro and Neuquén Provinces, Argentina (Fig. 1;^22^). A focal study population for each species was established in 1992 on Estancia Rincon Grande (*C. sociabilis*) and Estancia San Ramon (*C. haigi*); these study sites are located a few hundred meters from one another, on opposite sides of the Rio Limay^24^.

Despite their close geographical proximity, the two species differ markedly with respect to social structure, demography, and genetic variation. *Ctenomys sociabilis* is group living, with burrow systems routinely shared by multiple adults^25^. This species has been characterized by low mitochondrial genetic diversity over the past several millennia^14^, including an abrupt decrease in genetic variation approximately 3,000 years ago concordant with an eruption of the Puyehue-Cordón Caulle volcanic complex^26^. In contrast, *C. haigi* is solitary, with each adult inhabiting its own burrow system^28^; this species has historically been characterized by higher levels of mitochondrial genetic diversity than *C. sociabilis* ^14^. Given these differences in genetic variation and the extensive pre-eruption data sets for these species, comparative studies of *C. sociabilis* and *C. haigi* should offer important insights to the immediate genomic impacts of a marked reduction in population size.

### Sample collection

Pre-eruption tissue samples were collected during December 2001; post-eruption samples were collected during December 2011 and again in December 2012-January 2013. In all cases, live capture of individuals, collection of non-destructive tissue samples, and release of individuals were performed following the methods of Lacey *et al*.^11,27,28^. All field procedures were approved by the UC Berkeley Animal Care and Use Committee and followed the guidelines established by the American Society of Mammologists for the use of wild mammals in research^29^.

Tissue samples from 57 *C. sociabilis* were analyzed. Seventeen of these were pre-eruption samples from the focal population at Rincon Grande while 31 were collected post-eruption (December 2011) from the same population. No individual was included more than once in our analyses of genetic variation. To minimize potential kinship among the individuals examined, the pre-eruption samples were chosen to represent 13 different burrow systems, the majority of which (76.5%) were sampled only once. In contrast, the 31 post-eruption samples were from 9 burrows; of these, 6 of these burrows had multiple residents included in our post-eruption dataset. To add spatial perspective to our analyses, post-eruption samples were also obtained from 9 additional individuals from a second population of *C. sociabilis* at Estancia La Lonja, located about 3 km to the northwest of the focal Rincon Grande population. The La Lonja population had not been sampled prior to the eruption. Due to logistic challenges imposed by the substantive ash fall in the region, this was the only non-focal population of *C. sociabilis* that we were able to access following the eruption. Sampling of the focal study population of *C. haigi* at San Ramon consisted of 12 pre-eruption and 17 post-eruption (December 2012–January 2013) samples, with no material obtained from additional populations of this species.

### DNA extraction

DNA was extracted from all tissue samples using the Qiagen DNEasy Blood and Tissue extraction kits. Extractions were performed immediately prior to the preparation of genomic libraries (see below) to minimize the possibility of sample contamination. Following extraction, DNA sample concentrations were measured using a Qubit fluorometer (Qubit dsDNA HS assay, Life Technologies) and Nanodrop (ThermoFisher). Samples with low yields were re-extracted using a ZR Genomic DNA Tissue Miniprep kit (Zymo); this process, followed by ethanol precipitation, increased the concentrations of DNA in these extracts.

### Genomic library preparation

DNA libraries for targeted gene capture were prepared following the dual index protocol of Kircher *et al*.^30^, as modified by Crawford^31^. In brief, 580 ng of DNA from each individual was sheared using a BioRuptor Sonicator (Diagenode), after which a small quantity of the resulting fragmented DNA was visualized on a 1.2% agarose gel to verify fragment size distribution. Sonication was performed for four rounds at 90-second intervals to optimize fragment sizes of 450 bp. We then performed blunt-end repair using T4 DNA polymerase as per Meyer and Kircher^32^, after which reactions were cleaned using Ampure XP beads. We then ligated P5 and P7 adapters to each fragment and performed adapter fill-in with Bst polymerase. Following this, we measured DNA concentration using a Nanodrop and then conducted two rounds of indexing PCR reactions (11 cycles each) using a Phusion High Fidelity kit and standard Illumina indices. Every sample was then divided into two pools, each of which was indexed separately with a unique combination of P5 and P7 indices in order to reduce errors during sequencing^30^. We used experimental assays of indexing PCRs with different numbers of cycles, followed by analysis of the resulting fragment length distribution with a Bioanalyzer assay, to determine the number of cycles per reaction that would minimize the number of PCR duplicates and thus maximize read mapping^33^. Once indexing PCRs were complete, we quantified DNA concentrations again using Nanodrop, and then performed automated size selection (Pippen Prep, Sage Science) to isolate DNA fragments ranging in size from 580 to 610 bp. Fifty nanograms of each size-selected library were then aggregated in to a single pool in preparation for the targeted gene capture.

### Targeted gene capture and sequencing

Custom in-solution probes were designed using the NimbleGen SeqCap EZ Target Enrichment System; the details of this procedure are given in Crawford (2016)^31^. We identified 2,027 target segments of nuclear DNA based on analyses of *C. sociabilis* and *C. haigi* transcriptomes^31^. Targeted regions were approximately 1,200 bp long and putatively neutral. Capture of these target regions was completed using the NimbleGen SeqCap protocol for hybridization, with several modifications. In brief, we performed an in-solution hybridization of our pooled libraries with the custom probes. After incubation at 47 °C for 70 hours, we washed and recovered the captured multiplex DNA sample using M-270 Streptavidin Dynabeads. We then amplified the captured DNA via ligator-mediated PCR using the HiFi HotStart ReadyMix (KAPA Biosystems). We performed three parallel rounds of ligator-mediated PCR, checking the resulting DNA concentrations after each PCR reaction (using Qubit) and adjusting the number of cycles in subsequent reactions in order to reduce PCR stochastic drift^34^. The first PCR reaction was performed for 13 cycles, while the second and third PCR reactions were run for 14 cycles. Amplification products were then pooled in equimolar amounts, cleaned with Ampure XP beads, and subjected to Bioanalyzer analysis to gauge the concentration and distribution of product sizes. Finally, to assess the success of hybridization, we performed a series of quantitative PCR reactions using five different primer pairs: three primer pairs had been designed to amplify regions targeted by our probes (MATR F/R: 5′TCCTAGTCTCAACCCAGTGCT 3′/5′GTTATGCGAGGTCTCACCAA 3′; MR1 F/R: 5′AATGTGGCTCTCATCACCAA 3′/5′AATCTCTTGAGCCAGGCAAT3′; RNF130 F/R: 5′TGGATTGCCTTGCTACAGAG 3′/5′TTGTGACTGGCTCCTCTTTG3′), while the remaining two primer pairs had been designed to amplify “control” regions not targeted by these probes (Mat2A F/R: 5′TTGTGGATACTTATGGCGGTT3′/5′AAGAACCCTCCTGCACAGAC3′; CS1 F/R: 5′CCTGGCAAGTGTACTTCCGT/5′ GTCAGCAGGGTCAATCCAGT 3′). These qPCR reactions allowed us to quantify the rate of enrichment for target sequences, thereby confirming that our capture procedure was successful prior to submitting samples for sequencing. Finally, samples were sequenced with 300 bp paired-end reads on an Illumina HiSeq. 2500 RAPID platform at the UC Berkeley QB3 core facility; our samples were pooled with those from other projects for a total of 240 samples run across three sequencing lanes.

### Sequence cleanup, assembly, and variant calling

We cleaned and verified the quality of the Illumina sequences obtained following the protocol of de Wit *et al*.^35^. Specifically, we used the FastX toolkit^36^ to trim and remove regions of sequence with quality scores below 20, after which we removed reads that were <20 bp in length. Quality distributions were visualized using FastQC^37^ and Galaxy^38^. We then merged all reads for the same individual using custom Bash and Python scripts, after which we used Bowtie2^39^ to align these merged reads to the original transcriptome sequences used to design our bait capture probes. Following alignment, reads were processed with SAMtools^40^, which generated summary statistics for average sequence depth and cover for each individual. Based on these distributions, we used a custom Python script and SNPCleaner^41^ to further filter the sequences to ensure high confidence in our reads. Using these scripts, we allowed a maximum of one mismatched base per read and applied quality thresholds derived from those used by Bi *et al*.^34^ for working with both historical and modern DNA. Specifically, at the individual level, we eliminated any individuals with extremely low or high coverage (less than one-third or more than three times the average coverage across all individuals). We then filtered out reads below the 5^th^ or above the 95^th^ percentiles of coverage depth for all samples, which was approximately 3X and 100X, respectively. Finally, we removed sites with a root mean square mapping quality below 10. Single nucleotide polymorphisms (SNPs) in the remaining reads were identified using the software package ANGSD^42^.

### Population genomics analyses

We generated multiple summary statistics to assess post-eruption changes in genetic variation. Prior to these analyses, we used LOSITAN^43^ to identify and remove any loci that appeared to be under directional selection. This program, which uses an F_ST_-outlier method to identify signs of selection, was run for 50,000 simulations with a false discovery rate of 0.1. Additionally, we used a custom Python script to identify and remove any loci that were not in Hardy-Weinberg equilibrium. Collectively, these procedures ensured that the variation contained in our final data set reflected genome-wide consequences of demographic changes driven by neutral processes, rather than changes driven by directional selection.

### Genetic differences among populations

To quantify genetic variation within populations, we used a custom Python script to calculate mean heterozygosity for each population sampled. To account for potential differences in heterozygosity resulting from the variable number of individuals sampled per population, we conducted a bootstrap analysis in which we subsampled an equal number of randomly selected individuals from each population; this process was repeated 100 times, after which we calculated mean heterozygosity for each population based on these subsamples. Similarly, to minimize the potential effects of loci with extremely high or low levels of heterozygosity, we randomly selected a subsample of 100 loci and then estimated heterozygosity based on this panel of SNPs; this process was repeated 100 times, after which mean heterozygosity was calculated across all iterations. To examine additional measures of genetic diversity, we plotted per-site distributions of Watterson’s theta (θ_W_) and nucleotide diversity (π) for each population. Finally, to determine if estimates of genetic diversity were influenced by the demographic structure of the population, we partitioned the data by sex and calculated mean heterozygosity for randomly selected subsets of males and females. For *C. sociabilis*, we also calculated mean heterozygosity and inbreeding coefficients using data from only a single randomly selected individual per burrow system in both pre- and post-eruption populations.

Next, we examined patterns of genetic diversity among populations using ngsTools^44^ and custom Python and R scripts. First, we calculated pairwise F_ST_ values for all populations of conspecifics. Then, to assess differentiation among populations visually, we generated principal component analyses (PCA) plots for both species using a custom Python script. We also generated PCA plots in which data for each species were subdivided by 1) sex, 2) age (juvenile or adult), and 3) burrow system (for *C. sociabilis*) to determine if any of those factors influenced estimates of genetic differentiation among populations. As another measure of genetic differentiation among populations, we conducted admixture analyses using a range of values for K, the number of putative populations. We ran these analyses using the *a priori* hypotheses of K = 2 (accounting for the two spatially distinct populations) and K = 3 for *C. sociabilis* (accounting for the three populations sampled that were either temporally or spatially distinct), and K = 2 for *C. haigi* (accounting for the two populations sampled that were temporally distinct). We also explored outcomes for values up to K = 6 for both species to determine if a greater number of putative populations revealed unanticipated evidence of genetic differentiation.

### Effects of number of loci examined

To explore the effects of the number of loci analyzed on estimates of genetic variation, we used a custom Python script to conduct a simulation in which we varied the number of loci used to characterize variation in the pre- and post-eruption populations of *C. sociabilis* from Rincon Grande, as well as the post-eruption population of this species from La Lonja. We randomly subsampled loci, starting with *n* = 50 and incrementing by 50 loci per iteration to a total of *n* = 1000. Following this, we then subsampled *n* = 1000 to *n* = 10,000 loci in 500 locus increments. In these simulations, subsampling was repeated 100 times for each value of *n*, with observed heterozygosity determined for each subsample. We then calculated the mean and associated standard deviation for estimates of heterozygosity obtained for each value of *n*.

### Inferring signals of demographic history

To identify demographic processes that may have contributed to changes in pre- versus post-eruption patterns of genetic variation, we used custom Python scripts to generate folded site frequency spectra (SFS) for each species. SFS provide an overview of the distribution of allelic frequencies, which can reveal evidence of past demographic events such as reductions in population size^45–47^. Because differences in the numbers of individuals or alleles sampled can affect such distributions, we conducted SFS analyses using equal numbers of randomly selected pre- and post-eruption individuals; this subsampling was repeated 20 times for each time period, after which allele frequency distributions were averaged across iterations and pre- and post-eruption data sets were compared for each focal study population.

Because patterns of allele sharing among populations may also reflect demographic factors such as bottlenecks, we determined the number of private alleles – alleles unique to the populations in which they were detected – for each population sampled, as well as the number of alleles shared between two, but not all three, of the populations of *C. sociabilis* sampled. For both sets of analyses, we completed 100 iterations of random subsampling with equal numbers of individuals per population to control for any effects due to differences in the actual number of individuals genotyped per population. In addition, to control for any potential effects due to sampling of multiple individuals per burrow system in *C. sociabilis*, we conducted 100 iterations of subsampling in which only one randomly chosen individual per burrow system was included in each population. To confirm that these analyses were not biased by outlier loci, we also randomly subsampled 100 loci; we repeated this procedure 100 times, after which we assessed the mean occurrence of private and shared alleles across iterations.

To explore further potential signals of demographic change related to the volcanic eruption, we plotted distributions of Tajima’s D for each population sampled. This statistic, which can reflect both past demographic events and selection, was estimated across 100-bp sliding window regions using ngsTools; use of a distribution of values for Tajima’s D allows for more robust assessments of demographic history than a single mean estimate of this parameter^48,49^. We also generated pairwise mismatch distributions and their associated raggedness indices^50^ for each population using a custom Python script. The shapes of mismatch distributions can provide insights into recent demographic history, while raggedness indices can be used to compare such distributions quantitatively^51^. Finally, to determine if levels of inbreeding changed after the eruption, we calculated coefficients of inbreeding (F) for each individual using Plink^52^, after which we calculated mean values of f for each pre- and post-eruption population.

### Demographic simulations

To explore the potential impacts of demographic processes on post-eruption genetic variation in our study populations, we conducted a series of simulations. First, to quantify the expected effects of drift resulting from post-eruption declines in population size, we simulated an approximately 50% reduction in each of our pre-eruption study populations; this level of decline was largely consistent with that observed for *C. sociabilis* immediately following the 2011 eruption^16^. Specifically, we randomly designated 50% of individuals in each pre-eruption sample as survivors, after which we calculated heterozygosity for both the entire pre-eruption population and for the “surviving” post-eruption population. This procedure was repeated 100 times to generate a mean and standard deviation for heterozygosity in the simulated populations. Although this procedure did not take into account other demographic processes that may have affected post-eruption genetic variation, it provides a general estimate of expected changes in post-eruption heterozygosity due solely to stochastic loss of individuals from a population.

To examine the potential impacts of other demographic processes on genetic variation, we used MIGRATE-N^53^ to examine post-eruption data from the Rincon Grande and La Lonja populations. This program allows the testing of different models of demographic history by generating Bayes Factors, derived from marginal likelihoods for different historical scenarios. We tested the following four hypotheses regarding post-eruption population structure at Rincon Grande and La Lonja: (1) panmixia (2) bi-directional movement (immigration and emigration) between the populations, (3) immigration only (La Lonja to Rincon Grande), and (4) emigration only (Rincon Grande to La Lonja). Although the power of our analyses may have been somewhat limited due to the assumption of constant population sizes and the failure to incorporate observed post-eruption demographic changes, tests of these hypotheses provide an important first step toward determining whether different patterns of migration may have shaped genetic variation in post-eruption populations of *C. sociabilis*.

## Results

Our sequence alignment resulted in a mean per-individual depth of coverage of 11.46 for *C. sociabilis* and 11.41 for *C. haigi*, with greater than 99.9% cover per individual of targeted areas for both species. No individuals had less than one-third or greater than three times the mean coverage, indicating that all animals fell within the suggested thresholds for filtering samples with extremely low or high coverage^34^. After examining all SNPs for evidence of departures from Hardy-Weinberg equilibrium, we excluded two sites from analyses for *C. sociabilis* and six sites from analyses for *C. haigi*. Similarly, we excluded an additional 24 loci for *C. sociabilis* and 4 for *C. haigi* that were identified by LOSITAN as potentially being under directional selection. Thus, the final data set consisted of 531 SNPs for *C. sociabilis* and 449 SNPs for *C. haigi*. Despite the overall greater number of variant sites analyzed for *C. sociabilis*, the mean number of variant sites per individual for this species (N = 16.35) was less than that for *C. haigi* (N = 34.55).

### Pre- and post-eruption genetic differentiation

Mean heterozygosity in both focal study populations was reduced following the 2011 eruption. In *C. sociabilis*, mean heterozygosity across all sites decreased from 0.00167 pre-eruption to 0.00130 post-eruption; for *C. haigi*, these values were 0.00310 and 0.00274, respectively. For both species, this difference in variation was significant (two sample Kolmogorov–Smirnov test; both p < 0.0001). The same outcome was obtained when comparing estimates of pre- and post-eruption heterozygosity for a randomly selected subsample of 100 loci per species (*C. sociabilis*: pre = 0.00164, post = 0.00131; *C. haigi*: pre = 0.00273, post = 0.00231; two sample Kolmogorov-Smirnov tests, both p < 0.0001), indicating that this outcome was not impacted by interspecific differences in the number of loci examined. This pattern of post-eruption reduction in genetic variation was also evident in the distributions of the per-site values of θ_W_ and π, both of which showed a shift toward lower values after the eruption (Supplemental Figs 1 and 2).

A reduction in post-eruption variation was also detected for *C. sociabilis* for subsamples based on one randomly chosen individual per burrow system (pre = 0.00169; post = 0.00124; two sample Kolmogorov-Smirnov test, p < 0.0001). In contrast, there was no apparent difference in heterozygosity between males and females in the post-eruption population of this species at Rincon Grande (males: 0.00130, females: 0.00129; two sample Kolmogorov-Smirnov test, p < 0.0001); this was the only population of *C. sociabilis* for which sample sizes were sufficient for comparisons of known-sex individuals. Among populations of *C. sociabilis*, post-eruption heterozygosity at La Lonja (0.00122) was significantly lower than either pre- or post-eruption heterozygosity at Rincon Grande (two sample Kolmogorov-Smirnov test, both p < 0.0001). Pre-eruption samples from La Lonja were not available for analysis and thus we could not determine if the lower post-eruption variation in this population reflects consistent differences between La Lonja and Rincon Grande or if the impacts of the 2011 eruption were greater at La Lonja.

**Figure 2:**
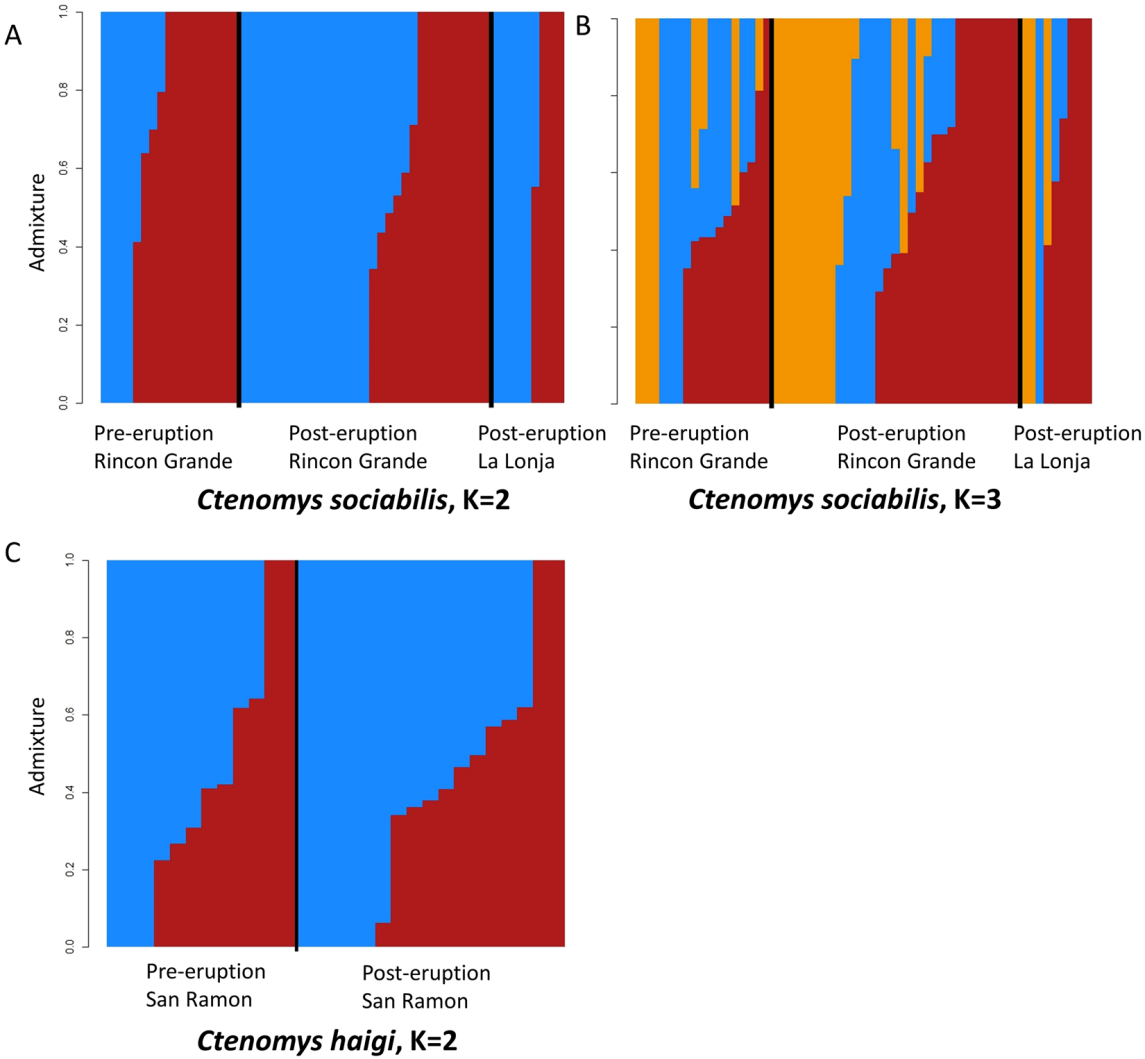
Admixture plots for *C. sociabilis* and *C. haigi*. For each panel, the populations and temporal periods (pre- versus post-eruption) examined are indicated. For *C. sociabilis*, the number of putative populations was set at (**A**) K = 2 and (**B**) K = 3. For *C. haigi* (**C**), analyses were run with K = 2.

The relatively modest reductions in heterozygosity detected for both study species were associated with limited evidence of genetic differentiation between the pre- and post-eruption samples from these animals. For example, F_ST_ values across time periods were low for both *C. sociabilis* (0.00348) and *C. haigi* (−0.00042), suggesting little temporal differentiation between pre- and post-eruption populations. Similarly, principal component analyses failed to reveal strong temporal differentiation in either study species (Supplemental Fig. 3). Further, admixture plots for *C. sociabilis* (Fig. 2A and B) provided no clear indication of temporal segregation between populations of this species; outcomes did not differ as the number of putative populations varied from K = 3 to K = 6. Similarly, admixture analyses revealed no evidence of temporal differentiation between pre- and post- eruption samples of *C. haigi* (Fig. 2C). Thus, although genetic variation was reduced in both study species, the 2011 eruption of Puyehue-Cordón Caulle complex did not appear to result in immediate temporal differentiation of the pre- and post-eruption populations.

### Effects of number of loci examined

**Figure 3:**
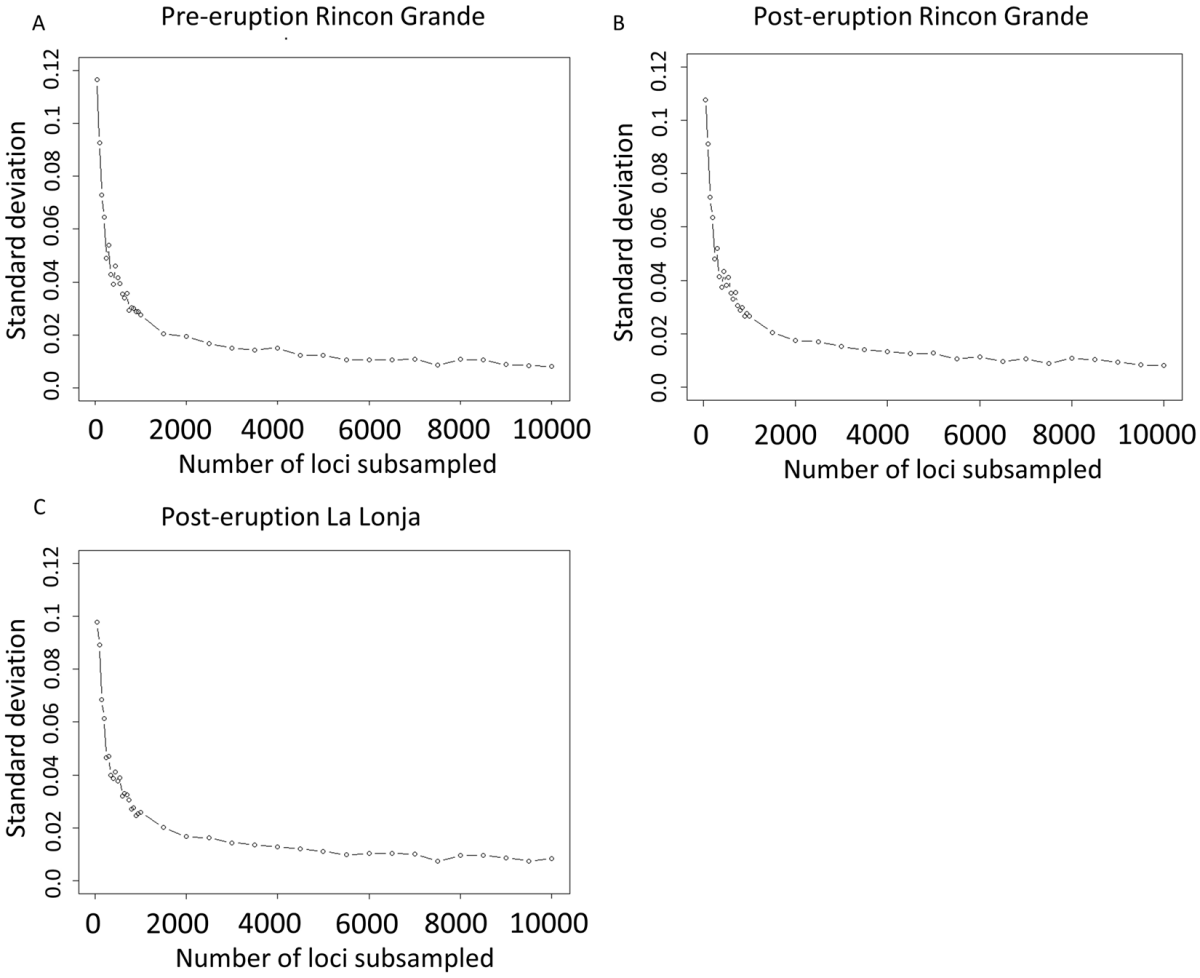
Relationship between the number of randomly subsampled loci (100 iterations each) and the standard deviation of mean heterozygosity. Data are shown for three *C. sociabilis* populations: (**A**) pre-eruption Rincon Grande, (**B**) post-eruption Rincon Grande, and (**C**) post-eruption La Lonja.

Varying the number of loci used to characterize genetic variation revealed that increasing the number of loci subsampled reduced the standard deviations around estimates of mean heterozygosity (Fig. 3). Indeed, there was a sharp decline in standard deviation as the number of loci examined increased from the initial value of 50 to approximately 1,000 loci, after which the impact of number of loci on estimates of standard deviation of mean heterozygosity appeared to stabilize.

### Signatures of demographic processes

Folded site frequency spectra (SFS; Supplemental Fig. 4) were similar in shape for pre- and post-eruption populations of both study species, providing no evidence of recent differences in demographic history between *C. sociabilis* and *C. haigi*. Subsampling of the data set to control for differences in the numbers of individuals and alleles examined resulted in a slight post-eruption increase in the proportion of low-frequency polymorphisms in each species, contrary to expectations following a reduction in population size. These changes, however, were not significant (two sample t-tests; p > 0.05 for both species), and thus SFS analyses failed to reveal evidence of post-eruption changes in demography.

**Figure 4:**
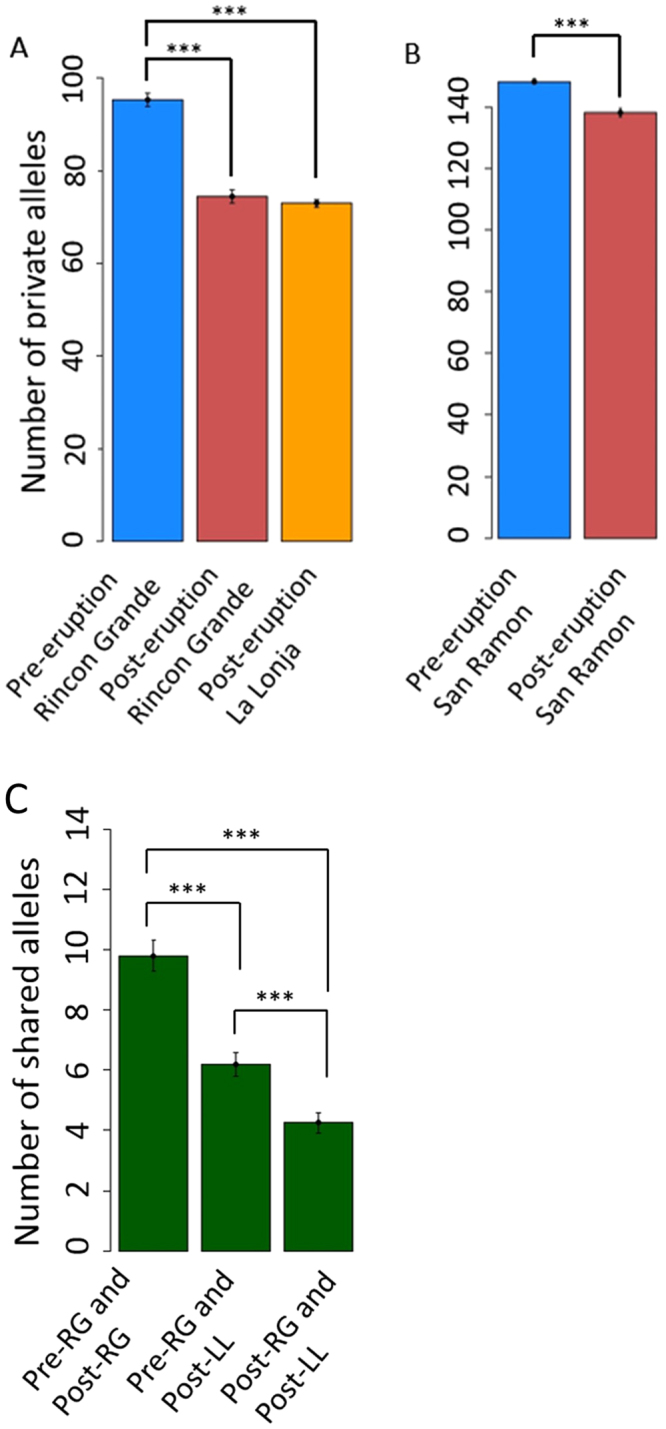
Comparisons of the mean (±SE) number of private alleles per population for (**A**) *C. sociabilis* and (**B**) *C. haigi*. For both species, values for pre- and post-eruption populations are indicated. In (**C**), the number of all alleles shared between two (but not three) populations is shown for all pairwise combinations of populations of *C*. *sociabilis*. Populations are abbreviated as following: pre-eruption Rincon Grande (Pre-RG), post-eruption Rincon Grande (Post-RG), and post-eruption La Lonja (Post-LL). ***Indicates significant contrasts (all p < 0.001).

Within the focal population of each study species, the number of private alleles differed significantly between pre- and post-eruption samples (two-sample T test; both p < 0.0001). In both species, post-eruption populations were characterized by fewer private alleles (Fig. 4), suggesting that one consequence of the reported reductions in population size was the loss of unique alleles. We also compared the distribution of alleles shared between any two (but not all three) populations of *C. sociabilis*. Of the three pairwise comparisons conducted (pre- and post-eruption Rincon Grande; pre-eruption Rincon Grande and post-eruption La Lonja; and post-eruption Rincon Grade to post-eruption La Lonja), the pre- and post-eruption Rincon Grande populations shared the greatest number of alleles, while the post-eruption Rincon Grande and post-eruption La Lonja populations shared the fewest alleles (Fig. 4C; two-sample T tests; p < 0.0001 for each pairwise comparison). The same general result was obtained for comparisons conducted using (1) a randomly selected subset of individuals per population, (2) a subsample consisting of only one randomly chosen individual per burrow system, and (3) a randomly selected subset of loci. These findings indicate that overall patterns of allele sharing among populations of *C. sociabilis* were not biased by differences in the number of animals or number of loci sampled per population.

**Figure 5:**
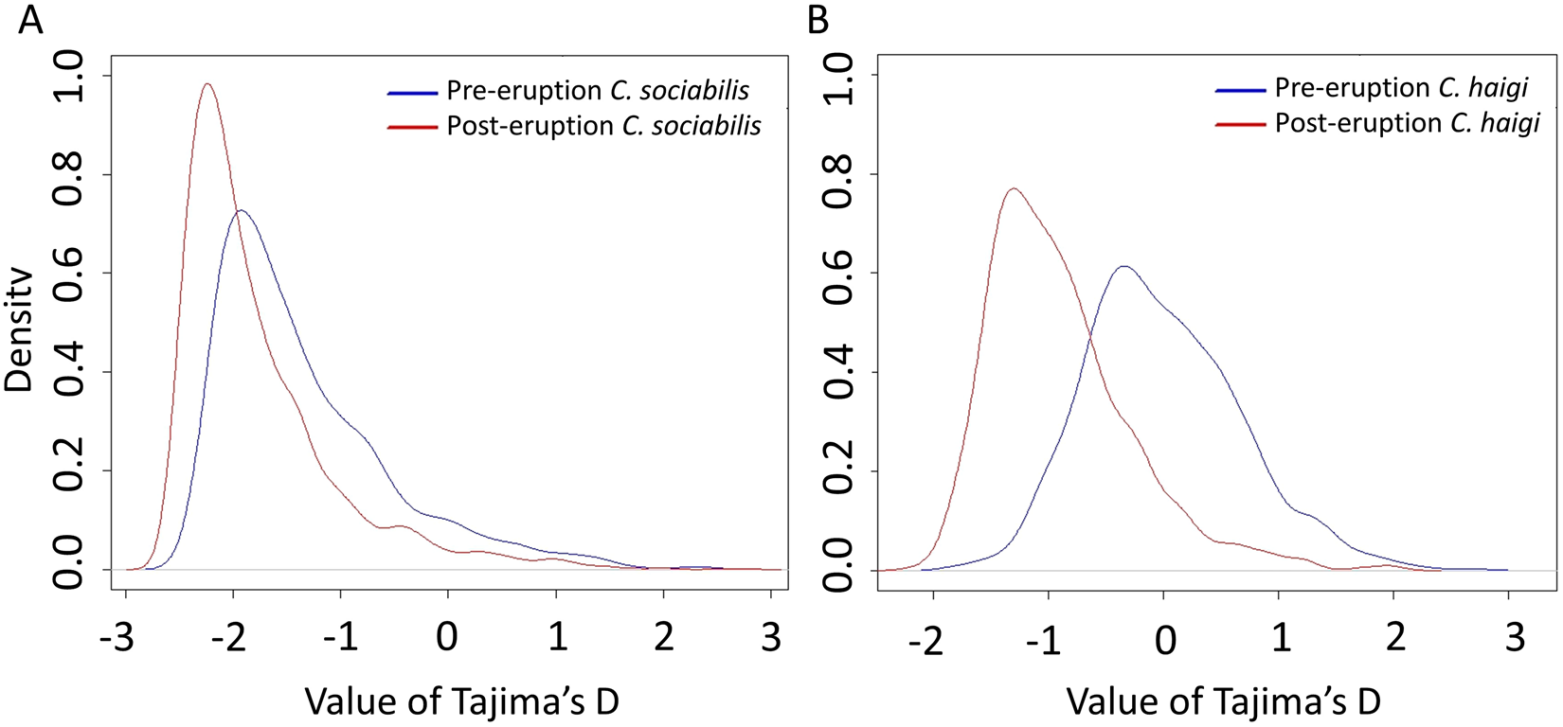
Smoothed kernel density distribution of values of Tajima’s D for (**A**) *C. sociabilis* and (**B**) *C. haigi* based on analyses of 100-bp sliding windows. Density denotes the relative frequency of individual values of Tajima’s D across the 100-bp sliding windows.

Both pre- and post-eruption values of Tajima’s D (calculated across 100-bp sliding windows) were generally negative for each study species, indicating an excess of low frequency alleles. Contrary to expectation, modal values of D decreased following the eruption in both study species (mean change from pre- to post-eruption *C. sociabilis:* −0.429; *C. haigi*: −0.892; two sample Kolmogorov–Smirnov tests; both p < 0.0001; Fig. 5), suggesting that low frequency alleles were more abundant after the 2011 eruption.

**Figure 6:**
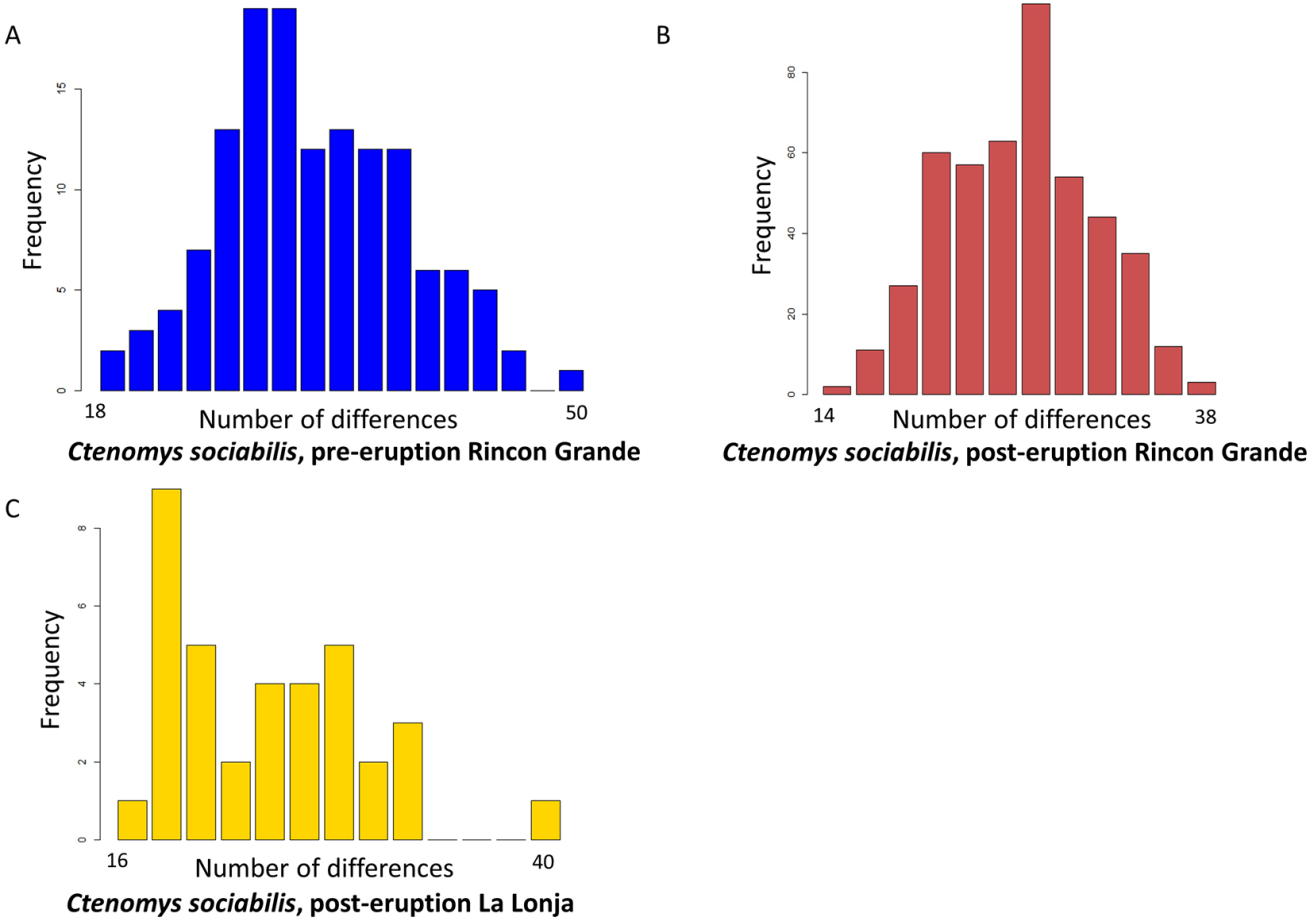
Pairwise mismatch distributions for *C. sociabilis* depicting the frequency of the number of sequence differences across all pairs of individuals in a given population: (**A**) pre-eruption Rincon Grande, (**B**) post-eruption Rincon Grande, and (**C**) post-eruption La Lonja.

**Figure 7:**
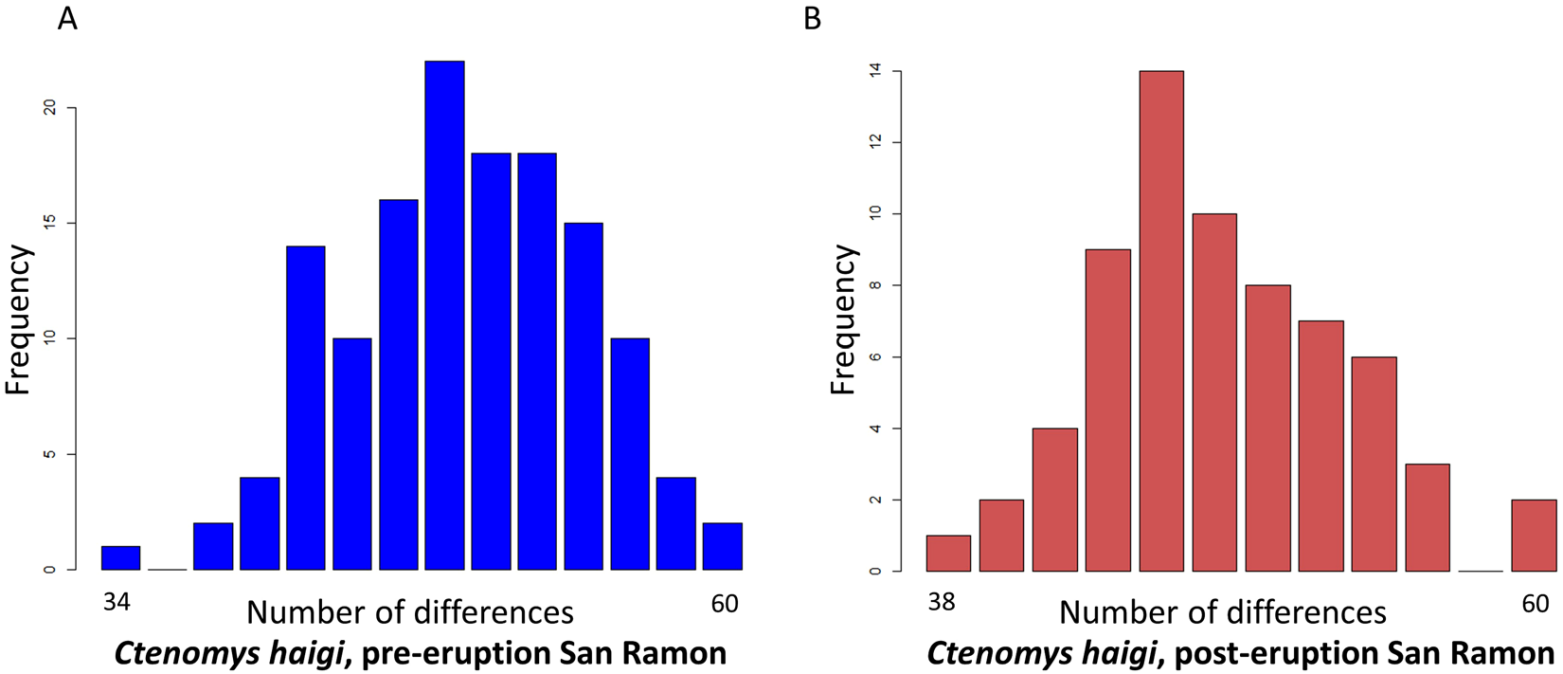
Pairwise mismatch distributions for *C. haigi* depicting the frequency of number of the number of sequence differences across all pairs of individuals in a given population: (**A**) pre-eruption San Ramon and (**B**) post-eruption San Ramon.

Inspection of mismatch distributions revealed that the pre-eruption focal populations of each study species were characterized by a single primary peak (Figs 6A and 7A), with Harpending’s raggedness index values of r = 0.0102 for *C*. *sociabilis* and r = 0.0156 for *C. haigi*. Post-eruption, mismatch distributions for these populations remained unimodal (Figs 6B and 7B) with raggedness indices of r = 0.0244 for *C. sociabilis* and r = 0.0230 for *C. haigi*. In contrast, the mismatch distribution for the post-eruption samples of *C. sociabilis* at La Lonja was more ragged, with an index value of r = 0.0887 (Fig. 6C). No significant change in mismatch distributions, however, was detected for either species when comparing the centered, re-scaled mismatch distributions from pre- and post-eruption populations (two sample Kolmogorov-Smirnov test; both p > 0.05). Collectively, these analyses provide no clear evidence of recent demographic changes in either study species.

Finally, mean values for individual coefficients of inbreeding (F) in the focal population of *C. sociabilis* at Rincon Grande were greater after the eruption, even when the data were restricted to one randomly subsampled individual per burrow system (pre-eruption: −0.204, post-eruption: 0.127; two-sample t-test, *p* < 0.0001). The mean value of F for the post-eruption population of *C. sociabilis* at La Lonja was −0.0143, which fell within the range of values reported for this species at Rincon Grande. In the absence of pre-eruption samples from La Lonja, we could not assess the impacts of the eruption on levels of inbreeding in this population. There was no significant difference between pre- and post-eruption values of F in the focal population of *C. haigi* (pre-eruption, 0.159; post-eruption, 0.0580; two-sample t-test, p = 0.087). Thus, our analyses suggest that the 2011 eruption resulted in increased inbreeding within the focal study population of *C. sociabilis* but not the focal study population of *C. haigi*.

### Simulations of demographic parameters

The simulated 50% reduction in size of the pre-eruption population of *C. sociabilis* at Rincon Grande did not result in a significant change in heterozygosity (pre: 0.03337; post: 0.03338; two-sample T test, *p* = 0.29). Our Bayesian modeling analyses revealed that the most strongly supported demographic hypothesis was that of panmixia between the Rincon Grande and La Lonja populations (model probability = 0.986), with the hypotheses of full migration (model probability = 0.00467), immigration from La Lonja to Rincon Grande (model probability = 0.00667), and emigration from Rincon Grande to La Lonja (model probability = 0.00271) receiving considerably less support. Thus, we found little evidence to suggest that post-eruption changes in genetic variation in *C. sociabilis* were influenced by migration between populations.

## Discussion

Our results indicate that in both *C. sociabilis* and *C. haigi* the reduction in population size documented immediately following the 2011 eruption of the Puyehue-Cordón Caulle volcanic complex^16^ was associated with a decrease in genetic variation, as revealed by measures of mean heterozygosity across the multiple loci examined. In the more extensively sampled *C. sociabilis*, this decrease in genetic variation was evident throughout the focal population, with no apparent differences in response to the eruption based on the sex of the animals or the number of burrow systems sampled. Despite the post-eruption decrease in heterozygosity detected in each study species, we found little evidence of temporal or spatial genetic differentiation between populations of conspecifics, suggesting that the eruption did not lead to increased genetic structure. Because these results are based on analyses of a large number of putatively neutral markers distributed throughout the genome, we believe that our findings are indicative of the impact of the eruption on genetic variation in the study animals. Intriguingly, the decrease in genetic variation reported here for *C. sociabilis* contradicts previous findings regarding the impacts of the Puyehue-Cordón Caulle eruption; our prior analyses based on a much more limited suite of microsatellite loci^16^ had revealed an increase in post-eruption genetic variation in the focal study population for this species. This difference in outcomes raises intriguing questions regarding the abilities of different types of molecular markers, including associated differences in numbers of loci surveyed, to detect the genetic consequences of recent demographic changes.

### Loss of genetic variation following population decline

Classical population genetics theory suggests that reductions in genetic variation associated with demographic bottlenecks are dependent on the magnitude and duration of the reduction in population size^34,54,55^. Due to the relatively modest declines in population size (~50%) observed^16^ and the short duration of this decline (<2 generations) prior to the collection of our post-eruption samples, we had not expected to find significant changes in post-eruption genetic variation in the focal study populations. As noted above, however, previous analyses based on microsatellite loci^16^ revealed a significant post-eruption increase in genetic variation in *C. sociabilis*, an unexpected result that, based on demographic modeling, appeared to reflect post-eruption changes in migration and gene flow. In contrast, the data set considered here revealed small but significant decreases in genetic variation in both study species, a finding that is more consistent with theoretical expectations (e.g. Nei *et al.* ^1^). Given the considerably larger molecular data sets employed in this study, we expect that the reductions in post-eruption genetic variation reported here are more indicative of the genetic changes experienced by our study populations.

### Factors affecting post-eruption genetic variation

Although reductions in population size are typically expected to lead to declines in genetic variation^1^, the exact processes by which such declines occur may vary. We found little evidence to suggest that selection was acting on the regions of the genome targeted for analysis. Very few of the loci examined (<5%) revealed departures from Hardy-Weinberg expectations or signals of directional selection. Further, because loci demonstrating departures from neutral expectations were excluded from analyses of genetic variation in our study populations, we do not believe that the post-eruption changes in variation reported here resulted from strong selective forces acting on these animals.

Similarly, it is unlikely that the observed post-eruption changes in genetic variation were the result of altered mutation rates following the eruption. Although mutation rates can be affected by external factors (e.g. Ellegren *et al*.^56^), we are not aware of any demonstrated or suggested links between ash produced by volcanic eruptions and increases in genomic mutation rates. Further, the average rate of mutation for mammalian genomes has been estimated at 2.2 × 10^−9^ per base pair per year^57^. Thus, given the limited temporal interval between our pre- and post-eruption sampling, this rate of mutational change could not have produced the observed post-eruption changes in genetic variation.

Although Hsu *et al*.^16^ reported increases in post-eruption genetic variation and suggested that increased migration and gene flow contributed to these changes, our analyses failed to reveal evidence of significant changes in these demographic parameters following the 2011 eruption. Populations at equilibrium^58^ as well as populations which have undergone a recent bottleneck^51,59–61^ are expected to display a multimodal mismatch distribution with relatively high values of raggedness. In contrast, pairwise mismatch distributions for both the pre- and post-eruption focal populations of *C. sociabilis* and *C. haigi* were roughly unimodal with low levels of raggedness, findings that are more typical of populations that are expanding or that are characterized by high levels of immigration and gene flow^50,62,63^. More importantly, these distributions were not significantly different between pre- and post-eruption samples, providing no evidence of demographic change in this interval. Thus, the results of our mismatch analyses suggest that (1) neither focal study population was in demographic equilibrium prior to the 2011 eruption and (2) the eruption did not substantially alter patterns of migration and gene flow in these populations.

### Comparisons of molecular markers

Our analyses offer important insights into the genomic consequences of an abrupt reduction in size in natural populations of mammals. While apparent population bottlenecks have been reported for other species of *Ctenomys* based on analyses of microsatellite and allozyme data^16,64–67^, our study offers the first direct comparison of pre- and post-bottleneck samples using genomic-level data. Accordingly, our analyses employed a much larger number of loci to detect changes in genetic variation. The presumably greater resolution offered by our data set is important given the relatively modest reductions in size and density detected in our focal study populations following the 2011 eruption. While studies of other ctenomyid species have examined the genetic consequences of decreases in population size of >90%^26,64,65^, our focal study populations experienced declines of only ~25–50%, suggesting that associated changes in genetic diversity would also be relatively modest. As a result, the use of a large panel of markers located throughout the genome should have increased our likelihood of detecting genetic signatures of these reductions in population size.

Particularly intriguing is the contrast between our results and those of Hsu *et al*.^16^, who reported a post-eruption increase in microsatellite heterozygosity in *C. sociabilis*. Although the same post-eruption samples were used in both studies, the pre-eruption samples examined differed: the samples analyzed here were collected in 2001, while those genotyped by Hsu *et al*.^16^ were collected during 1993–1998. It is possible that this difference in data sets contributed to the contrasting outcomes for these studies. However, given the generally low levels of genetic variation in both sets of pre-eruption samples (see also Lacey^11^), it seems unlikely that the temporal difference between these data sets underlies the reported differences post-eruption changes in genetic variation. Indeed, a marked reduction in population size in 1999 (Lacey, pers. comm.) should have reduced pre-eruption variation among the individuals sampled here compared to those analyzed by Hsu *et al*.^16^.

Instead, we posit that this difference in outcomes is due primarily to the greater power of our genomic-level markers to detect modest differences in genetic variation arising over short time periods. The number of markers employed in this study was more than two orders of magnitude greater than that used by Hsu *et al*.^16^ and this difference alone is likely to have affected the reliability of the post-eruption estimates of genetic diversity generated by each study^2^. Consistent with this, our analyses of the effects of number of loci on measures of genetic diversity revealed that the variance in estimates of mean heterozygosity decreased markedly as the number of loci employed increased, suggesting that larger numbers of these markers generate more robust metrics of genetic variation. Consequently, studies relying on a small number of markers likely show greater variance and more potential for summary statistics that are not representative of the true variation across the genome. In addition, microsatellite markers have been found to be unreliable for estimating genomic diversity, with marked differences in estimates of genetic variation generated with microsatellites versus SNPs^68,69^. Further, microsatellites have been found to have limited ability to detect complex demographic histories, including bottlenecks followed by rapid population growth^70^, and models demonstrating consistent tempo and mode in the evolution of microsatellites are lacking^71^. These limitations, combined with the presumptively greater analytical power of a large panel of genomic markers leads us to expect that estimates of genetic variation based on the latter provide a more accurate picture of pre- versus post-eruption changes in genetic variation in our study populations. If this supposition is correct, our analyses indicate that both *C. sociabilis* and *C. haigi* experienced decreases in genetic variation following the 2011 volcanic eruption.

## Conclusion

Our analyses of genetic variation in two species of ctenomyid rodents impacted by the 2011 eruption of the Puyehue-Cordón Caulle volcanic complex indicate that abrupt, naturally occurring reductions in population size can impact genetic variation over even brief time scales. For both *C. sociabilis* and *C. haigi*, analyses of genome-wide markers revealed small but significant reductions in post-eruption heterozygosity. Multiple lines of evidence supported the hypothesis that these reductions in genetic variation were associated with post-eruption declines in population size, indicating that even modest (~50%) reductions occurring over a span of 1–2 generations are sufficient to generate signals in genomic variation. Characterizing the impacts of such demographic changes has critical implications for understanding and for predicting how species will respond genetically to altered environmental conditions and thus may generate important insights into the conservation of species affected by such events.

## Electronic supplementary material

Supplemental figures
