# Supplementary material for: Genomic data reveal a loss of diversity in two species of tuco-tucos (genus Ctenomys) following a volcanic eruption
Source: Sci Rep. 2017 Nov 24;7:16227. doi: 10.1038/s41598-017-16430-1 (PMC5701162; doi:10.1038/s41598-017-16430-1)
Supplement: Supplementary file 1 — Supplemental figures [file 41598_2017_16430_MOESM1_ESM.pdf]

Supplemental figures and information for "Genomic data reveal a loss of diversity in two species of tuco-tucos (genus *Ctenomys*) following a volcanic eruption"

Jeremy L. Hsu, Jeremy C. Crawford, Mauro N. Tammone, Uma Ramakrishnan, Eileen A. Lacey, and Elizabeth A. Hadly

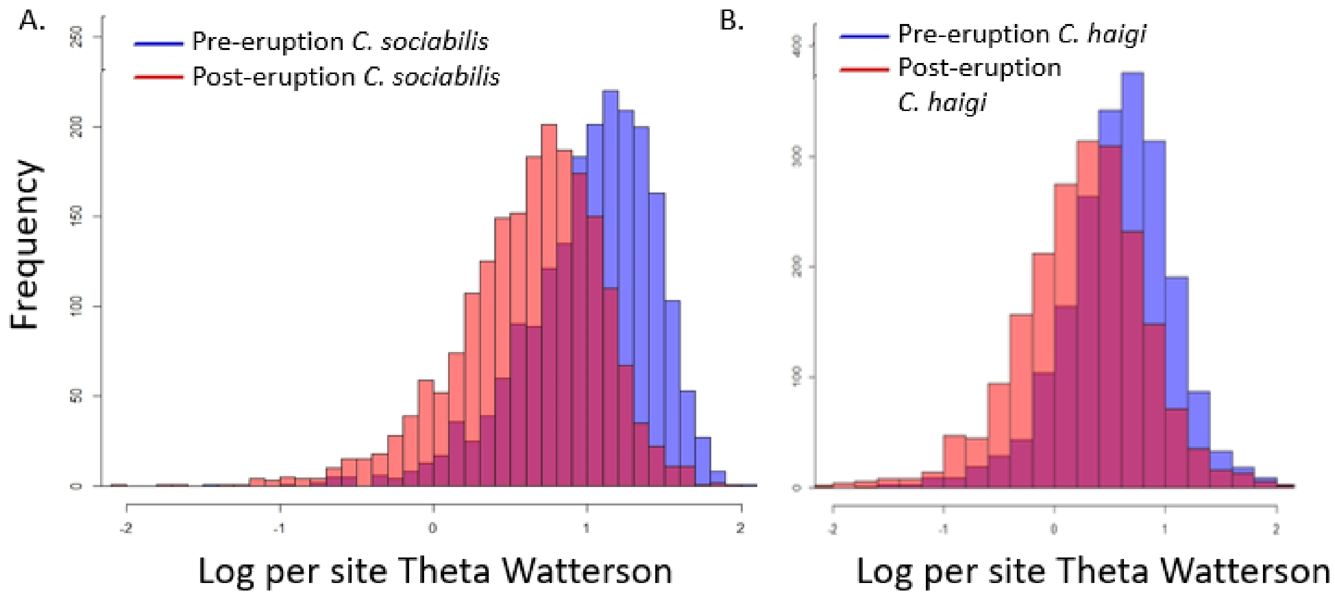

Supplemental figure 1. Distribution of global per-site values for Watterson's theta for **(A)** *C. sociabilis* and **(B)** *C. haigi*. Values are log-transformed and depict distributions of values for the focal population of each species for pre-eruption (in blue) and post-eruption (in pink) samples. Overlap between the two temporal distributions is shown in purple.

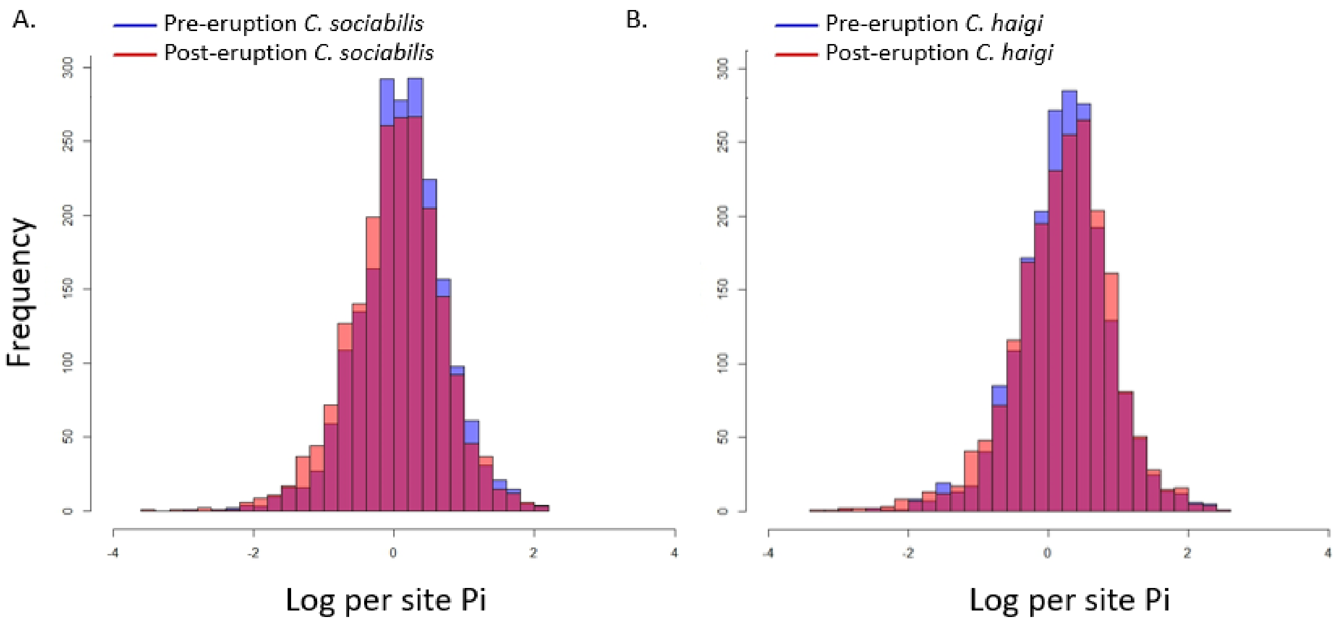

Supplemental figure 2. Distribution of global per-site values for pi (nucleotide diversity) for **(A)** *C. sociabilis* and **(B)** *C. haigi*. Values are log-transformed and depict distributions of values for the focal populations of each species for pre-eruption (in blue) and post-eruption (in pink) samples. Overlap between the two temporal distributions is shown in purple.

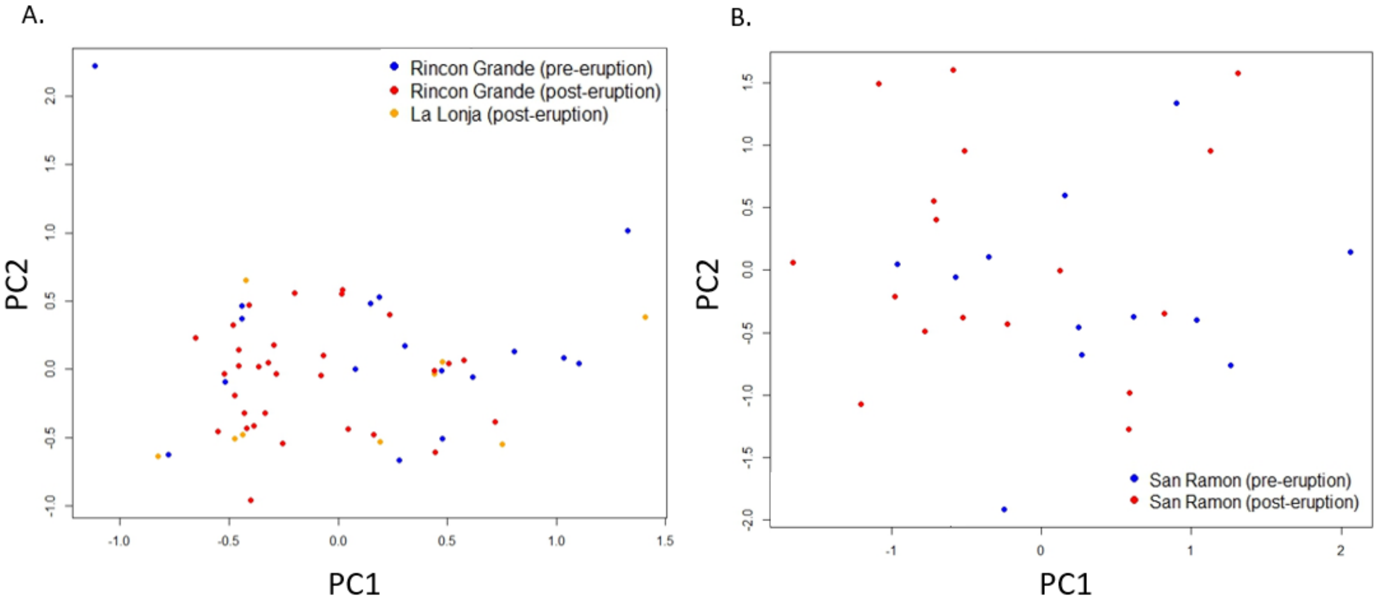

Supplemental figure 3. Plots of principal component analyses of genetic variation in **(A)** *C. sociabilis* and **(B)** *C. haigi*. PC1 and PC2 accounted for 4.9% and 4.1% of variation, respectively, for *C. sociabilis*, and 6.7% and 6.2%, respectively, for *C. haigi*.

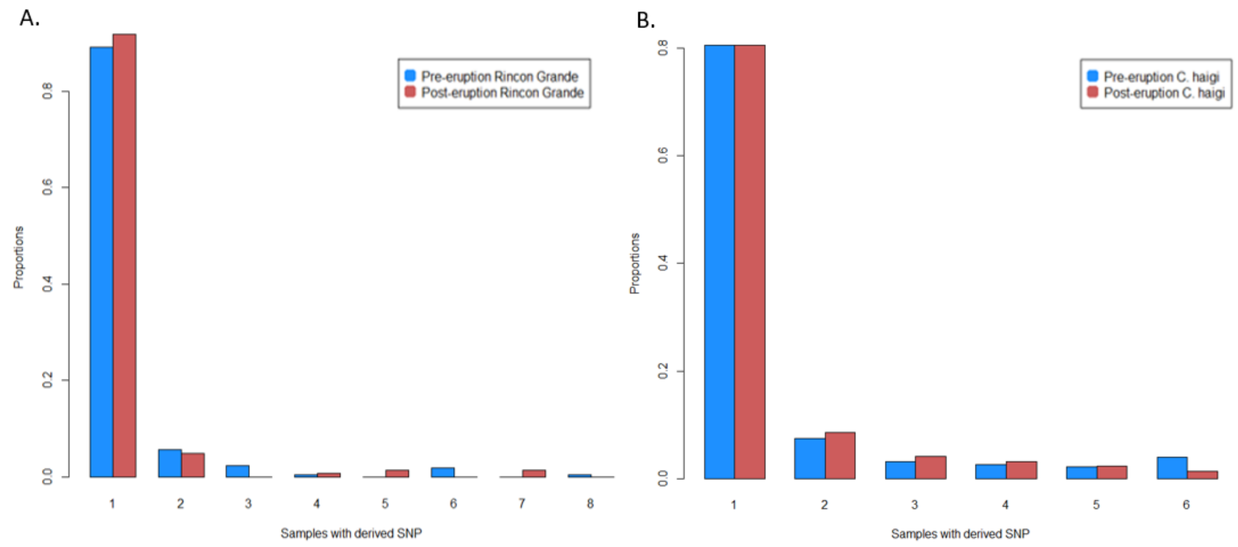

Supplemental figure 4. Comparisons of site frequency spectra for pre- and post-eruption focal populations of **(A)** *C. sociabilis* and **(B)** *C. haigi* using scaled, proportional values of allele frequencies.

Supplemental data. Spreadsheet of specimens included in study.

List of individuals sampled for analyses of pre- and post-eruption genetic variation. For each study species, the population or origin is indicated, as is the relative age (adult or juvenile) and the year of collection. The field identificatior number for each individual is also provided; because analyses were based on non-destructive tissue samples collected as part of long-term capture-mark-recapture studies, voucher specimens for these samples were not deposited in a museum collection.

	Species	Population	Individual ID number	Sex	Age	Year of collection	Burrow system
**Post-eruption**	*C. sociabilis*	Rincon Grande	4729296A3B (1)	Female	Juvenile	2011	ACENT
	C. sociabilis	Rincon Grande	5339240218 (2)	Male	Juvenile	2011	ACENT
	C. sociabilis	Rincon Grande	472825365F (3)	Male	Juvenile	2011	ACENT
	C. sociabilis	Rincon Grande	46360A1D55 (4)	Female	Adult	2011	OA
	C. sociabilis	Rincon Grande	5341DA5232 (5)	Male	Juvenile	2011	F
	C. sociabilis	Rincon Grande	472A2E7441 (6)	Female	Juvenile	2011	OA
	C. sociabilis	Rincon Grande	47247A1001 (7)	Male	Juvenile	2011	OA
	C. sociabilis	Rincon Grande	472E0D0649 (8)	Female	Adult	2011	ACENT
	C. sociabilis	Rincon Grande	53393C5307 (9)	Male	Juvenile	2011	OA
	C. sociabilis	Rincon Grande	47246B7419 (10)	Male	Juvenile	2011	AEXN
	C. sociabilis	Rincon Grande	471F1B0265 (11)	Male	Juvenile	2011	AEXN
	C. sociabilis	Rincon Grande	Male Juvenile (12)	Male	Juvenile	2011	ACENT
	C. sociabilis	Rincon Grande	472E15017F (13)	Male	Juvenile	2011	OA
	C. sociabilis	Rincon Grande	450761026A (14)	Female	Adult	2011	MIDMALLIN
	C. sociabilis	Rincon Grande	4724762104 (15)	Male	Juvenile	2011	ACENT
	C. sociabilis	Rincon Grande	472D79322B (16)	Female	Juvenile	2011	FINT
	C. sociabilis	Rincon Grande	447902125C (17)	Female	Juvenile	2011	FN1
	C. sociabilis	Rincon Grande	472A452620 (18)	Female	Juvenile	2011	F
	C. sociabilis	Rincon Grande	447B607D0B (19)	Female	Juvenile	2011	ACENT
	C. sociabilis	Rincon Grande	471F217527 (20)	Male	Juvenile	2011	OA
	C. sociabilis	Rincon Grande	47256F140B (21)	Female	Juvenile	2011	F
	C. sociabilis	Rincon Grande	47242E1C5C (22)	Female	Juvenile	2011	AEXN
	C. sociabilis	Rincon Grande	447F254C70 (23)	Female	Adult	2011	AEXN
	C. sociabilis	Rincon Grande	47261B1971 (24)	Female	Juvenile	2011	ANE
	C. sociabilis	Rincon Grande	47282F2D40 (25)	Male	Juvenile	2011	FN1
	C. sociabilis	Rincon Grande	472A5D7D4D (26)	Female	Adult	2011	AN-NE
	C. sociabilis	Rincon Grande	47243E182A (27)	Female	Juvenile	2011	AEXN
	C. sociabilis	Rincon Grande	46555F3843 (28)	Male	Juvenile	2011	ANE
	C. sociabilis	Rincon Grande	4635650403 (29)	Female	Juvenile	2011	OA
	C. sociabilis	Rincon Grande	447F2F2C3A (30)	Male	Juvenile	2011	FN1
	C. sociabilis	Rincon Grande	4724562204 (31)	Female	Juvenile	2011	FN1
**Pre-eruption**	*C. sociabilis*	La Lonja	LL1	Male	Juvenile	2010	NA
	C. sociabilis	La Lonja	LL2	Female	Adult	2010	NA
	C. sociabilis	La Lonja	LL3	Male	Juvenile	2010	NA
	C. sociabilis	La Lonja	LL4	Male	Juvenile	2010	NA
	C. sociabilis	La Lonja	LL5	Female	Adult	2010	NA
	C. sociabilis	La Lonja	LL6	Female	Adult	2010	NA
	C. sociabilis	La Lonja	LL7	Male	Juvenile	2010	NA
	C. sociabilis	La Lonja	LL8	Male	Juvenile	2010	NA
	C. sociabilis	La Lonja	LL9	Male	Juvenile	2010	NA
**Pre-eruption**	*C. sociabilis*	Rincon Grande	CS-535	Male	Juvenile	2001	PepsiLt
	C. sociabilis	Rincon Grande	CS-502	Female	Juvenile	2001	G
	C. sociabilis	Rincon Grande	CS-482	Male	Juvenile	2001	FN2
	C. sociabilis	Rincon Grande	CS-424	Female	Juvenile	2001	EC1
	C. sociabilis	Rincon Grande	CS-511	Male	Juvenile	2001	EC0.5
	C. sociabilis	Rincon Grande	CS-570	Female	Juvenile	2001	ASS-1
	C. sociabilis	Rincon Grande	CS-543	Male	Juvenile	2001	AS
	C. sociabilis	Rincon Grande	CS-611	Male	Juvenile	2001	AN
	C. sociabilis	Rincon Grande	CS-540	Male	Juvenile	2001	AB
	C. sociabilis	Rincon Grande	CS-507	Male	Juvenile	2001	54
	C. sociabilis	Rincon Grande	CS-392	Female	Adult	2001	PepsiLt
	C. sociabilis	Rincon Grande	CS-895	Female	Adult	2001	ECO
	C. sociabilis	Rincon Grande	CS-436	Female	Adult	2001	ASS-2
	C. sociabilis	Rincon Grande	CS-385	Female	Adult	2001	AN
	C. sociabilis	Rincon Grande	CS-394	Female	Adult	2001	AN
	C. sociabilis	Rincon Grande	CS-836	Female	Adult	2001	FN1
	C. sociabilis	Rincon Grande	CS-380	Female	Adult	2001	54
**Post-eruption**	*C. haigi*	San Ramon	2	Male	Juvenile	2013	NA
	C. haigi	San Ramon	3	Female	Adult	2013	NA
	C. haigi	San Ramon	4	Female	Juvenile	2013	NA
	C. haigi	San Ramon	5	Female	Adult	2013	NA
	C. haigi	San Ramon	6	Female	Juvenile	2013	NA
	C. haigi	San Ramon	7	Male	Adult	2013	NA
	C. haigi	San Ramon	8	Female	Juvenile	2013	NA
	C. haigi	San Ramon	9	Female	Juvenile	2013	NA
	C. haigi	San Ramon	10	Female	Adult	2013	NA
	C. haigi	San Ramon	11	Male	Juvenile	2013	NA
	C. haigi	San Ramon	12	Female	Adult	2013	NA
	C. haigi	San Ramon	13	Male	Adult	2013	NA
	C. haigi	San Ramon	14	Female	Adult	2013	NA
	C. haigi	San Ramon	15	Female	Adult	2013	NA
	C. haigi	San Ramon	16	Female	Adult	2013	NA
	C. haigi	San Ramon	19	Female	Juvenile	2013	NA
	C. haigi	San Ramon	20	Female	Adult	2013	NA
**Pre-eruption**	*C. haigi*	San Ramon	CH-218	Male	Adult	2002	NA
	C. haigi	San Ramon	CH-222	Female	Adult	2002	NA
	C. haigi	San Ramon	CH-227	Female	Adult	2002	NA
	C. haigi	San Ramon	CH-228	Female	Adult	2002	NA
	C. haigi	San Ramon	CH-236	Male	Adult	2002	NA
	C. haigi	San Ramon	CH-237	Female	Adult	2002	NA
	C. haigi	San Ramon	CH-240	Female	Adult	2002	NA
	C. haigi	San Ramon	CH-242	Male	Adult	2002	NA
	C. haigi	San Ramon	CH-243	Female	Adult	2002	NA
	C. haigi	San Ramon	CH-220	Male	Juvenile (#4)	2002	NA
	C. haigi	San Ramon	CH-221	Male	Juvenile (#11)	2002	NA
	C. haigi	San Ramon	CH-238	Male	Juvenile (#10)	2002	NA
